# Automatic Classification of Thyroid Findings Using Static and Contextualized Ensemble Natural Language Processing Systems: Development Study

**DOI:** 10.2196/30223

**Published:** 2021-09-21

**Authors:** Dongyup Shin, Hye Jin Kam, Min-Seok Jeon, Ha Young Kim

**Affiliations:** 1 Graduate School of Information Yonsei University Seoul Republic of Korea; 2 Healthcare, Life Solution Cluster, New Business Unit Hanwha Life Insurance Co Ltd Seoul Republic of Korea; 3 Data Analysis Team Aimmed Co Ltd Seoul Republic of Korea

**Keywords:** deep learning, natural language processing, word embedding, convolution neural network, long short-term memory, transformer, ensemble, thyroid, electronic medical records

## Abstract

**Background:**

In the case of Korean institutions and enterprises that collect nonstandardized and nonunified formats of electronic medical examination results from multiple medical institutions, a group of experienced nurses who can understand the results and related contexts initially classified the reports manually. The classification guidelines were established by years of workers’ clinical experiences and there were attempts to automate the classification work. However, there have been problems in which rule-based algorithms or human labor–intensive efforts can be time-consuming or limited owing to high potential errors. We investigated natural language processing (NLP) architectures and proposed ensemble models to create automated classifiers.

**Objective:**

This study aimed to develop practical deep learning models with electronic medical records from 284 health care institutions and open-source corpus data sets for automatically classifying 3 thyroid conditions: healthy, caution required, and critical. The primary goal is to increase the overall accuracy of the classification, yet there are practical and industrial needs to correctly predict healthy (negative) thyroid condition data, which are mostly medical examination results, and minimize false-negative rates under the prediction of healthy thyroid conditions.

**Methods:**

The data sets included thyroid and comprehensive medical examination reports. The textual data are not only documented in fully complete sentences but also written in lists of words or phrases. Therefore, we propose static and contextualized ensemble NLP network (SCENT) systems to successfully reflect static and contextual information and handle incomplete sentences. We prepared each convolution neural network (CNN)-, long short-term memory (LSTM)-, and efficiently learning an encoder that classifies token replacements accurately (ELECTRA)-based ensemble model by training or fine-tuning them multiple times. Through comprehensive experiments, we propose 2 versions of ensemble models, SCENT-v1 and SCENT-v2, with the single-architecture–based CNN, LSTM, and ELECTRA ensemble models for the best classification performance and practical use, respectively. SCENT-v1 is an ensemble of CNN and ELECTRA ensemble models, and SCENT-v2 is a hierarchical ensemble of CNN, LSTM, and ELECTRA ensemble models. SCENT-v2 first classifies the 3 labels using an ELECTRA ensemble model and then reclassifies them using an ensemble model of CNN and LSTM if the ELECTRA ensemble model predicted them as “healthy” labels.

**Results:**

SCENT-v1 outperformed all the suggested models, with the highest F1 score (92.56%). SCENT-v2 had the second-highest recall value (94.44%) and the fewest misclassifications for caution-required thyroid condition while maintaining 0 classification error for the critical thyroid condition under the prediction of the healthy thyroid condition.

**Conclusions:**

The proposed SCENT demonstrates good classification performance despite the unique characteristics of the Korean language and problems of data lack and imbalance, especially for the extremely low amount of critical condition data. The result of SCENT-v1 indicates that different perspectives of static and contextual input token representations can enhance classification performance. SCENT-v2 has a strong impact on the prediction of healthy thyroid conditions.

## Introduction

In South Korea, a large portion of medical services are maintained and operated under the public health insurance system [1-4], and the Korean National Health Insurance Corporation conducts biannual national health screening examinations. Apart from government-sponsored biannual health examination services, which are different from the health insurance system in the United States, Korean companies provide regular medical checkups to their employees annually according to Article 43 of the Occupational Safety and Health Act [5]. The entrusted companies conduct the examination in partnership with affiliated examination centers in large hospitals or professional examination centers and collect the results from individual medical institutions to provide follow-up health care services to the clients.

Electronic medical records (EMRs) and other forms of medical documentation are designed to focus on the convenience of work for medical personnel in line with the primary use of patient care. The text records of any examination numerical values and comprehensive findings provided by more than 1 examination institution are not standardized and are written in nonunified formats with different periods and health professionals. Thus, to ensure that consistent services are offered, a group of experienced nurses in examination work has been established using classification guidelines based on important keywords and by manually classifying individual test results to organize these results into a single unified format. In this study, thyroid ultrasonography and hormone tests were selected among the various measurements for the application of ensemble language models. The following sections are targeted for this study: individual text diagnosis of thyroid diseases, 3 numeric variables for thyroid hormone examination results, and comprehensive medical examination reports, including doctors’ comments.

When the rule-based text classification is considered for the analysis of contents in EMRs, repetitive classification and human labor–intensive verification can be required for an extensive rule set, regular expression, and branch logic because of a data model that is not designed for secondary usage of text data or sharing and interworking between multiple agencies [6-8]. However, various implementations in medical natural language processing (NLP) and applications of diverse language models can be considered with recent advances in NLP and techniques based on artificial neural networks [9-16] for data extraction, early detection of diseases, diagnostic support, and prediction of outcomes. Deep learning (DL) models represent intricate structures in large data sets by updating the internal parameters from backpropagation. Such learning techniques produce promising results in various tasks in processing images, videos, audio, and text data [17].

The data sets in our study are textual data that describe the findings and doctors’ comments from thyroid ultrasonography and additional comprehensive medical examination results. Such textual data can be considered and processed using NLP methods in DL. Referring to Wu et al [9], the most widely used DL model is recurrent neural network (RNN) variants, while Word2Vec [18] is the most common in embedding architectures. Among their reviewed papers, text classification has the highest percentage (41.5%) for clinical NLP tasks, followed by bidirectional encoder representations from transformers (BERT) [19]. BERT can be used by either training from scratch, directly using fixed pretrained models, or fine-tuning it.

In this study, we initially developed multiple single-architecture–based deep neural network models in NLP not only by using the efficiently learning an encoder that classifies token replacements accurately (ELECTRA) [20] model, which is a pretrained model with open Korean corpus data sets [21] in our study, but also by inventing a convolutional neural network (CNN) [22] model and long short-term memory (LSTM) [23] model. We chose the ELECTRA language model, which has an identical structure to BERT, because it achieves better performance on various NLP benchmarks than BERT and verifies that different pretraining methods are more effective for downstream NLP tasks. However, ELECTRA has a sequence limitation of 512 input tokens; thus, the LSTM structure is employed to capture the full length of contextual representations of input words. For the ELECTRA model, we propose a keyword-based trimming method for the comprehensive medical examination section of the input data sets to reflect thyroid-related information, which could be compulsively truncated because of limitations, effectively for the contextual representations.

**Figure 1:**
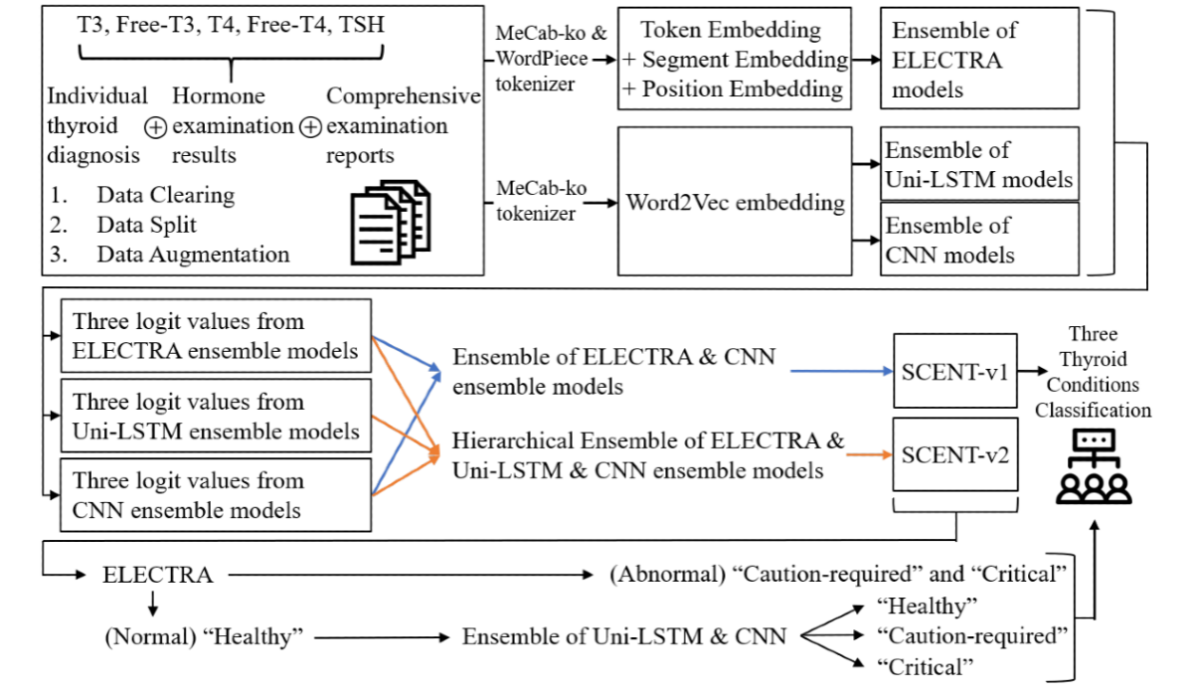
Overall flow of our proposed ensemble approach. T3: triiodothyronine; Free-T3: free triiodothyronine; T4: thyroxine; Free-T4: free thyroxine; TSH: thyroid stimulating hormone; ELECTRA: Efficiently Learning an Encoder that Classifies Token Replacements Accurately; Uni-LSTM: unidirectional long short-term memory; CNN: convolution neural network; SCENT: Static and Contextualized Ensemble NLP-neTworks; -v1: version 1; -v2: version 2.

Furthermore, we investigate and establish ensemble classification models based on the CNN, LSTM, and ELECTRA models. The combination of static and contextual NLP models is required not only to capture different perspectives of static and contextual word representations from the same input sequences but also to consider the characteristics of the data. The format of the data sets is not standardized or unified; thus, they can be prepared as complete sentences, lists of terminology-based words or phrases with or without numbering them, and groups of numerous medical examination measurements. Such aspects can be an obstacle, particularly for training the contextual relationships between input word tokens. Consequentially, we propose ensemble models to capture static and contextualized input word representations of textual examination data and classify them into 3 labels: healthy, caution required, and critical thyroid conditions. We construct 2 ensemble models and call them static and contextualized ensemble NLP network (SCENT) systems. SCENT version 1, SCENT-v1, is an ensemble or soft voting method for the CNN and ELECTRA ensemble models. SCENT-v2 is a hierarchical ensemble of CNN, LSTM, and ELECTRA ensemble models. SCENT-v2 initially classifies the 3 thyroid conditions using the ELECTRA ensemble model and reclassifies the selected labels, only if the ELECTRA ensemble model predicted them as “healthy” thyroid conditions, using an ensemble of CNN and LSTM ensemble models (Figure 1).

## Methods

### Data Labeling Using Thyroid Ultrasonography Keywords

Thyroid glands are butterfly-shaped endocrine glands located in the lower front of the neck and are responsible for the production of thyroid hormone [24]. Thyroid nodules are lumps produced by abnormal growth of thyroid cells that appear as either solid (hard lumps) or cystic (water lumps). If nodules are found in the thyroid gland during a medical examination, thyroid ultrasonography can be performed to check for signs of cancer. It is also possible to check thyroid hormone levels and conduct blood tests on thyroid antibodies to identify other types of thyroid disorders [25]. Thyroid nodules typically do not cause symptoms or require treatment, but a small number of thyroid nodules can be diagnosed as cancerous. Thyroid cancer is mainly detected and diagnosed using blood tests and thyroid ultrasonography. Thyroid ultrasonography may show the size and shape (solid or liquid-filled cysts) of thyroid nodules.

For our experimental data sets, to minimize classification errors, an experienced nurse with expertise in the field of health examination performed the first labeling task, and a member of another nurse group performed the second labeling of each entry. After that, reclassification proceeded through group discussions on the parts with differences in classification. In this study, the final classification tags for each entry were used as labels. The basic test results classification criteria are defined as follows:

- Healthy: no abnormalities (normal), simple cyst, tubular cyst, thyroid resection (thyroidectomy), benign calcification.
- Caution required: hypothyroidism, unequal parenchyma, internal thyroid disease, thyroiditis, nodule, thyromegaly, hyperechoic lesion, hypoechoic lesion, hyperechoic nodules, hypoechoic nodules, cystic lesions.
- Critical: tumor, malignant, biopsy, fine-needle aspiration cytology.

### Data Preprocessing

The data sets, which consist of individual text diagnosis of thyroid diseases, comprehensive medical examination text reports including doctors’ comments, and 3 categorical variables for individual hormone examination results, were classified as healthy, caution required, and critical labels in total. The categories of hormone examination results were classified as normal or abnormal by comparing the results of the numerous subtests for triiodothyronine (T3), free triiodothyronine (Free T3), thyroxine (T4), free thyroxine (Free T4), and thyroid-stimulating hormone with the reference range for each device and test. A total of 122,581 textual data were collected in the free form of EMRs from 284 health care institutions in the Republic of Korea between January 2015 and May 2020; thus, data clearing was compulsory. The data sets were written in Korean with numerous English biological and chemical terminologies, including various special characters. Many special characters and measurement units with brackets such as “blood pressure 120/80 mm/Hg”, “microalbuminuria is less than 30 mg/g”, and “renal cyst (left side 1.4 cm)” can increase vocabulary size and lengthen the sequence of input texts unnecessarily. Therefore, Korean, English, numerical characters, and only selected special characters, such as “%”, “'”, “/”, “~”, “²”, “-”, “,”, and “.” remained after preprocessing. In addition, the 3 dummy variables of hormone examination were converted concisely into 3 sentences before tokenization: “hormone examination results were normal,” hormone examination results were abnormal,” and “hormone examination was not conducted.”

Among the total sample size of 122,581 text data, 84,111 samples, 37,220 samples, and 1250 samples were labeled as healthy, caution required, and critical conditions, respectively. The extreme data imbalance can be troublesome for training or fine-tuning the DL models, so the least amount of critical condition data was initially divided into 7:1:2 ratios for training, validation, and test data sets. The training data were then augmented by splitting sentences and each sentence was attached one by one starting from the first sentence to the last. For instance, a sample datum with 3 consecutive sentences was multiplied into 3 samples with the first 1 sentence, the first 2 sentences, and the entire 3 sentences each from the original sample data. During the augmentation, the order of sentences was preserved as the original sample data because split sentences were added in the order of original sequences. Consequently, the critical condition data sets were split and then augmented, and the healthy and caution-required condition data sets were only divided according to the ratio of prepared data (Table 1). The training data sets for the critical condition were augmented from 875 to 29,174 samples. After that, the entire prepared training data sets were randomly shuffled. Relatively short examples of data and translations for each class are listed in Table 2. The data sets consist of a sequential combination of individual diagnosis, hormone examination results, and comprehensive medical examination reports. Comprehensive reports are occasionally omitted.

**Table 1:** Numbers of divided sample data sets. Only train data for critical thyroid condition are augmented and the original amount of data before the augmentation is given in brackets (N=122,581).

Thyroid conditions	Total number of prepared data sets				
	Train (n=87,524), n (%)	Validation (n=21,119), n (%)	Test (n=42,237), n (%)	Total, n (%)	
Healthy	29,175 (33.33)	18,312 (86.71)	36,624 (86.71)	84,111 (68.62)	
Caution required	29,175 (33.33)	2682 (12.70)	5363 (12.70)	37,220 (30.36)	
Critical	29,174 [875] (33.33)	125 (0.59)	250 (0.59)	1250 (1.02)	

**Table 2:** Short examples and English translations for each thyroid condition.

Examples			Contents
**Healthy condition**			
	Original	정상. 호르몬 검사 수치 정상입니다. uibc 감소, 철 증가, 총 콜레스테롤 증가, glucose증가, 골다공증.	
	Translation	Normal. Hormone examination results were normal. UIBC decreases, iron increases, total cholesterol increases, glucose increases, osteoporosis.	
	Original	정상. 호르몬 검사 수치 미 판정입니다. 체중 관리에 주의 가 필요합니다. 총 콜레스테롤 수치가 높습니다. 중성지방수치가 높습니다. 저밀도 콜레스테롤 수치 가 높습니다 .	
	Translation	Normal. Hormone examination was not conducted. Please be aware of weight management. Total cholesterol level is high. Neutral fat level is high. Low-density lipoprotein cholesterol level is high.	
**Caution-required condition**			
	Original	갑상선염. 호르몬 검사 수치 정상입니다. b형 간염 항체 미 형성. 갑상선염. 고 음영 유방, 유방 양성 석회화 양측.	
	Translation	Thyroiditis. Hormone examination results were normal. Hepatitis B antibody not formed. Thyroiditis. Dense breast, positive calcification for both.	
	Original	갑상선염 의심 또는 치유 반흔. 호르몬 검사 수치 정상입니다. 양측 치밀 유방 2. 갑상선염 의심 또는 치유 반흔 3. 담낭 결석 및 콜레스테롤 용종 4. 위염 5. 자궁경부 염 6. a형간염 항체 없음.	
	Translation	Suspect thyroiditis or scars. Hormone examination results were normal. Dense breasts for both. 2. Suspect thyroiditis or scars 3. Gallstone and cholesterol polyps 4. Gastritis 5. Cervicitis 6. No antibody for hepatitis A.	
**Critical condition**			
	Original	갑상선 초음파 검사상 좌엽 결절 2.78 cm 소견입니다. 세침 흡인 세포검사를 받으시 길 권유합니다. 호르몬 검사 수치 미 판정입니다.	
	Translation	Thyroid ultrasonography shows 2.78 cm of left nodule. We recommend taking a fine needle aspiration cytology. Hormone examination was not conducted.	
	Original	갑상선 좌측부에 10.2mm 크기의 저 에코결절이 1개 있으며 감별 진단을 위해 세침검사로 확인 요망됨. 결론은 좌측 부 갑상선 결절. 요망 세침검사로 확인 및 의사와 상담 요망. 호르몬 검사 수치 정상입니다. 위장 조영촬영결과 유 소견입니다. 갑상선 초음파 검사 결과 유 소견입니다.	
	Translation	There is 10.2mm size of 1 hypoechoic nodule in left-sided thyroid and requires fine needle aspiration cytology for differential diagnosis. Left-sided thyroid nodule in the conclusion. Have consultations with doctors and confirm with fine needle aspiration cytology. Hormone examination results were normal. Blood sugar level before a meal is high. Upper gastrointestinography results were abnormal. Thyroid ultrasonography results were abnormal.	

### Tokenization

Korean is an agglutinative language and one of the morphologically rich [26] and typologically diverse [27] languages; a character is composed of consonants and vowels of the Korean alphabets in 3 positional forms: choseong (syllable onset), jungseong (syllable nucleus), and jongseong (syllable coda). The positional forms are displayed in the lexicographic order of Korean alphabets as follows:

Choseong: ㄱㄲㄴㄷㄸㄹㅁㅂㅃㅅㅆㅇㅈㅉㅊㅋㅌㅍㅎ

Jungseong: ㅏㅐㅑㅒㅓㅔㅕㅖㅗㅘㅙㅚㅛㅜㅝㅞㅟㅠㅡㅢㅣ

Jongseong: (None)ㄱㄲㄳㄴㄵㄶㄷㄹㄺㄻㄼㄽㄾㄿㅀㅁㅂㅄㅅㅆㅇㅈㅊㅋㅌㅍㅎ

One of the common challenges in text preprocessing for Koreans is the ambiguity of word spacing, unlike other languages. For example, an English phrase “Be able to do” is translated into a grammatically accurate Korean phrase “할 수 있다,” which has 2-word spaces. When not strictly aware of Korean orthography, it can also be written as “할수 있다” (Beable todo) with 1-word space or “할수있다” (Beabletodo) without any word space. Furthermore, various postpositions or particles, which means “helping words” in English, are immediately attached after nouns or pronouns without any white space. For instance, English phrases “I am” and “You and me” become “Iam” and “Youand me” in Korean phrases. This can make it difficult to decompose sentences into distinguishable morphemes; for example, the same noun(s) or pronoun(s) can be tokenized into multiple tokens, even if their actual meaning may not differ. Such inconsistent grammatical errors and unique grammatical aspects can cause the same expression of word-level texts to be tokenized into different tokens, which may result in difficulty in training NLP models.

To resolve such problems, we used the MeCab-ko [28] tokenizer, which was originally introduced as MeCab for Japanese morphological analysis by Kudo et al [29]. The variation for the Korean tokenizer yields good performance to handle such problems by reconstructing and unifying a grammatical structure with a relatively faster speed than other Korean tokenizers [30]. WordPiece [31-33], which was originally introduced for Japanese/Korean segmentation, was employed for the transformer [34] encoder–based models such as BERT and ELECTRA for various purposes. One of the major advantages is that it can increase the robustness against the out-of-vocabulary (OOV) problem with a relatively small vocabulary size by disassembling words into subword units using a given text corpus. Therefore, in this study, we used the combination of MeCab-ko and WordPiece to pretrain the thyroid text data sets to fine-tune the ELECTRA [21] model, which was pretrained with a Korean open-source corpus. The input sentences were initially identified and reconstructed into possible grammatical morphemes by MeCab-ko and then segmented into divisible subwords that maximize the log likelihood of a language model by WordPiece. For instance, “임상적으로,” which means “clinically” in English, can eventually be tokenized into “임상,” “##적,” “으로” as “clinic,” “##al,” and “ly,” where the last part was initially separated by MeCab-ko and the first and second parts were segmented by WordPiece after that.

The average and maximum lengths of the input sequence resulting from different tokenizers are listed in Table 3. All input tokens from every sample had right-skewed (positive skewness) distributions. To reduce the sequence length, especially for the ELECTRA model, which has a limitation of 512 tokens, only comprehensive examination reports were trimmed for every sample. Based on the first sentence containing the word “thyroid,” all subsequent sentences including 1 previous sentence were extracted and then recombined with individual text diagnosis of thyroid diseases and textualized hormone examination results. Original comprehensive text reports were used when the word “thyroid” does not exist.

**Table 3:** Comparison of different tokenizers and the numbers of input tokens.

Tokenizer	Average number of tokens				Maximum number of tokens		
	Train	Valid	Test	Train		Valid	Test
MeCab-ko	494.2	522.3	520.1	2227		2219	2240
WordPiece for BERT^a^	664.7	698.7	695.9	4096		2943	4171
WordPiece for ELECTRA^b^	564.9	596.3	593.7	2656		2472	2500
MeCab-ko and WordPiece	540.6	570.0	567.6	2608		2431	2435
MeCab-ko (trimmed^c^)	370.4	419.6	418.8	2162		2219	2216
MeCab-ko and WordPiece (trimmed^c^)	404.9	457.6	456.6	2365		2431	2412

^a^BERT: bidirectional encoder representations from transformers.

^b^ELECTRA: efficiently learning an encoder that classifies token replacements accurately.

^c^Trimmed: The data sets were trimmed based on the keyword “thyroid” in the comprehensive medical examination text part.

### Proposed Framework

#### Overview

**Figure 2:**
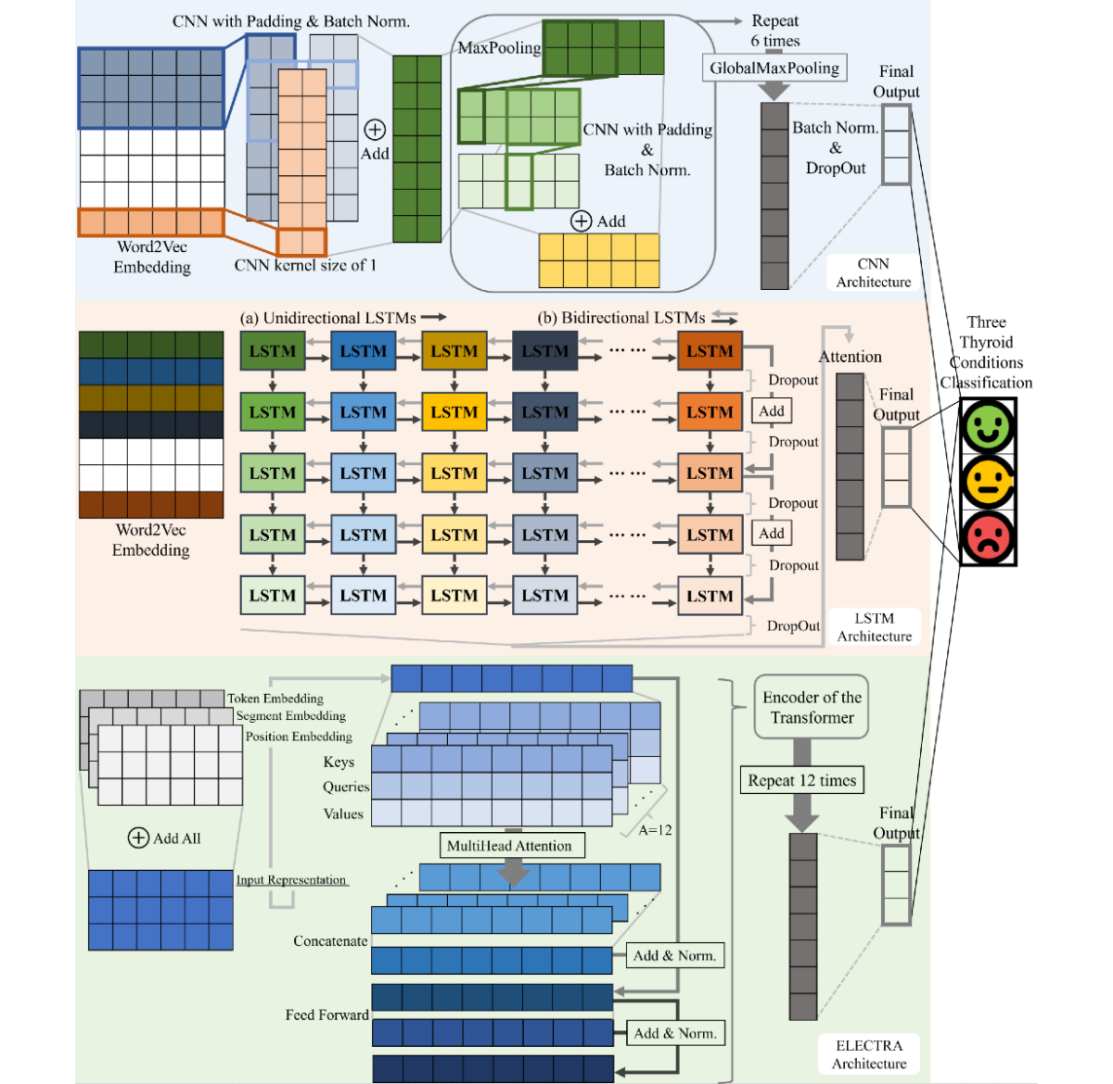
The architecture of the proposed ensemble models. Each model is trained or fine-tuned ten times for each ensemble model. Best viewed in color. CNN: convolution neural network; Batch Norm.: batch normalization transforms; LSTM: long short-term memory; ELECTRA: Efficiently Learning an Encoder that Classifies Token Replacements Accurately; Norm.: layer normalization.

In this study, we propose ensemble models, SCENT-v1 and SCENT-v2, which can reduce generalization errors of the prediction and reflect static and contextual perspectives of word representations in accordance with thyroid and general examination reports. Our proposed ensemble models consist of multiple single-architecture–based ensemble models from CNN, LSTM, and transformer encoder architectures as shown in Figures 1 and 2. We initially created a CNN with batch normalization (BN) [35] transform approach, LSTM with 2 shortcut connections [36] including an attention mechanism [37], and ELECTRA models. Each model was trained or fine-tuned 10 times with different settings of epochs, learning rates, and batch sizes. Subsequently, each single-architecture–based model was trained or fine-tuned 10 times and then combined into an ensemble model. In other words, 3 respective CNN, LSTM, and ELECTRA ensemble models were constructed by combining each of the 10 model’s prediction averages from softmax functions, or simply by performing soft voting, to stabilize the variances of classification performance. Based on the experimental results of single-architecture–based ensemble models, we selected the CNN-Word2Vec, Uni-LSTM, and ELECTRA-v2 ensemble models for further ensemble approaches. The 3 distinct models were CNN with trainable Word2Vec embedding, unidirectional LSTM with trainable Word2Vec embedding, and the second version of ELECTRA fine-tuned with trimmed data sets.

The final predictions for the thyroid condition classification were then determined using ensemble and hierarchical ensemble methods, namely, SCENT-v1 and SCENT-v2, respectively. In this experiment, static word representations were captured from the CNN-Word2Vec ensemble model, and contextualized word representations were captured from the Uni-LSTM ensemble model with the ELECTRA-v2 ensemble model, which exclusively considers the initial 512 token sequences in the trimmed data sets. SCENT-v1 is an ensemble of CNN and ELECTRA ensemble models, and SCENT-v2 is a hierarchical ensemble of CNN-Word2Vec, Uni-LSTM, and ELECTRA-v2 ensemble models (Figure 1). The multilabel classification in SCENT-v2 was based on the 3 thyroid condition predictions from the ELECTRA-v2 ensemble model and reclassified the selected labels using an ensemble of CNN-Word2Vec and Uni-LSTM ensemble models, where only the ELECTRA-v2 ensemble model predicted “healthy” thyroid conditions. In other words, SCENT-v2 kept the decisions from the ELECTRA-v2 ensemble model for “caution required” and “critical” thyroid condition predictions, and then made final decisions from an ensemble of CNN-Word2Vec and Uni-LSTM ensemble models only for the “healthy” thyroid conditions, which were predicted by the ELECTRA-v2 ensemble model.

**Figure 3:**
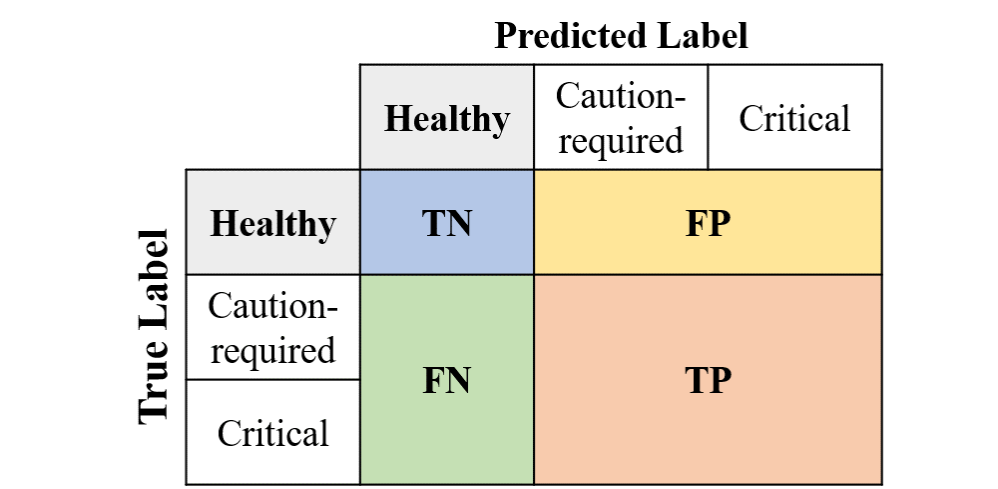
A confusion matrix for healthy thyroid condition datasets. TN: true negative; FP: false-positive; FN: false-negative; TP: true positive.

Our proposed SCENT-v2 is designed for the industrial purpose in that it saves time and cost by reducing the number of manual thyroid condition classification steps required and human misclassification errors. Perfect overall classification accuracy for current and future data sets must be the ideal solution. However, there are numerous obstacles such as imbalanced numbers of data sets and the difficulty level of the problem. This hierarchical ensemble method, therefore, was pursued to minimize the numbers of false negatives and maximize the numbers of true negatives as depicted in Figure 3. It primarily aimed to correctly predict an exceedingly high number of healthy thyroid conditions with 100% precision of healthy (negative) thyroid labels and leave the remaining data sets for manual classification to provide precise health care services and reduce the human classification workloads. This approach was proposed to take into account aspects of practical and industrial usage efficiency by sacrificing the overall accuracy but reducing the large manual workloads. Based on the validation data sets, among the 3 single-architecture–based ensemble models, the ELECTRA-v2 ensemble model indicates a relatively low number of false positives, and the 2 CNN-Word2Vec and Uni-LSTM ensemble models show a relatively small number of false negatives in the prediction of healthy thyroid conditions (Figure 3). Accordingly, we constructed SCENT-v2 for the hierarchical ensemble model in this study.

#### Embedding

Word embedding is a way of expressing words that are converted into distributed vector representations. Mikolov et al [18] introduced Word2Vec embedding, which provides remarkable performance for capturing syntactic and semantic word relationships. Continuous bag of words (CBOW) and Skip-gram methods were proposed with several loss function approaches in their paper, and we used skip-gram with negative sampling (SGNS) Word2Vec in our NLP models. For a given corpus sequence *T* length of words *w*_1_, ... , *w_t–_*_1_, *w_t_*, *w_t_*_+1_, ..., *w_T_*, where the training context size is *c*, CBOW predicts the probability of the current word *w_t_* as *P*(*w_t_|w_t-c_*, ..., *w_t+c_*). By contrast, the skip-gram method predicts the probability of the context words as *P*(*w_t–c_*, ..., *w_t–_*_1_, *w_t_*_+1_, ..., *w_t_*_+_*_c_|w_t_*) by the softmax function calculated as




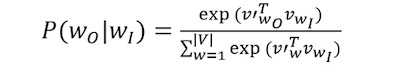




where *v_w_* and *vʹ_w_* are the input and output word vector representations, respectively; *|V|* is the vocabulary size; and *w_O_* and *w_I_* refer to the target word representations and the given word representations, respectively. Negative sampling is suggested as an alternative to the initially used hierarchical softmax function because of the cost of computing the vocabulary size. It is defined by the objective function calculated as




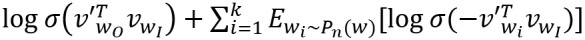




where every log*P*(*w_O_|w_I_*) in the objective is replaced. The probability *P_n_*(*w_i_*)=*f*(*w_i_*)^3/4^⁄∑*_j_*_=0_[*f*(*w_j_*)^3/4^] is a unigram distribution that allows the use of a selected number of *n* negative samples instead of the number of vocabulary sizes. To use the SGNS Word2Vec, we initially predefined 5 context sizes, 5 negative samples, and 300 dimensions for each vector representation. The embedding was then pretrained unsupervised using Wikipedia corpus data [38], which contain 162,861 articles on various topics. The grammatical expressions in the corpus data were restructured and prepared using the MeCab-ko tokenizer before pretraining. This method helps convert the *i*th word *w_i_* to a fixed length of 300-dimensional word vector *x_i_*, and thus, can be calculated algebraically; for instance, *vector(“한국_Korea_”)–vector(“서울_Seoul_”)+vector(“도쿄_Tokyo_”)* results in a vector representation with most similarity of the word *일본_Japan_* (the subscripts are English translation).

Word embeddings for transformer-based models BERT and ELECTRA have a different approach for establishing word vocabulary because of the tokenizer called WordPiece. Rather than the n-gram strategy in Word2Vec, this approach initializes the vocabulary with its size to include all character representations in each corpus by using a greedy longest-match-first [39] approximation, which picks the longest subwords or prefixes inside the corpus. It selects a new word piece that maximizes the log likelihood for the corpus when the word piece is added to the language model. For example, a word piece “un” is added to the vocabulary if the probability of “un” divided by “u” and “n” is higher than other subword units. After the preparation of token embeddings, both transformer-based models create their input representations by summing up the token, segment, and position embeddings. Two special tokens were used to distinguish sentences. A special classification token (CLS) was inserted as the first token of all sentences for the classification task, and a special separator token (SEP) was used to distinguish sentence pairs as the first and second sentences, where the segment embedding distinguishes them. The position embedding shows the location of each token as PE_(_*_p,_*_2_*_i_*_)_=sin(*p⁄*10000*^2i/d^*) and PE_(_*_p,2i_*_+1)_=cos(*p⁄*10000*^2i/d^*), where *p* indicates the location of the embedding vector in the input sentence; and *i* is the index of the dimension within the embedding vector. A hyperparameter *d*=768 indicates the dimensions of all corresponding embedding vectors and encoder layers of the transformer.

#### Convolution Neural Networks

CNN can be described as a structure that is originally designed for processing images to identify patterns of features by weight sharing and local connectivity. CNN can be used for NLP as well and extracts the same features regardless of positions by sliding CNN filters over consecutive tokens with a fixed window size. CNNs have become an essential method in computer vision tasks [40-42] and produce good results on sentence classification tasks [43]. In this study, we suggest deep CNN feature–learning methods to determine how static word vector representations are achieved in text classification. The model, which is depicted at the top of Figure 2, considers input word tokens through pretrained Word2Vec, where the maximum length of input sequences is set to 2240 tokens. The CNN model initially vectorizes input word tokens through word embeddings with a dimensionality of 300 for each vector representation. A convolution operation then generates a feature map *c*=*f*(*Wx*+*b*), where *W* and *b* are the weight and bias parameters of the model, respectively, and *f*(∙) is a nonlinear function such as rectified linear units [44] and ReLU(*x*)=max(0, *x*). We employed the BN transform in the convolutional operation before the nonlinearity function. In this study, we used the BN transform in the CNN operations because it [35] can reduce the necessity for dropout [45], and other methods such as *L*_2_ regularization become ineffective when combined with BN, but only influence learning rates [46].

Starting from the lower layers of the CNN model, we conducted the summation of 2 consecutive 3 kernel sizes of convolution layers with BN and 1 kernel size of the convolution layer without BN from pretrained SGNS Word2Vec. The word vectors with a dimensionality of 300 are represented as local features of word vectors with 250 dimensions. The structure then connects to a max-pooling combination consisting of size 3 and stride 2 of max-pooling, 2 consecutive 3 kernel sizes of convolution layers with BN, and a simple shortcut connection with a consistency of 250 dimensionality. The combination was repeated 6 times to determine deep representations of static word features, and a global max-pooling operation extracted the maximum values over the dimensions. The penultimate layer was then connected to the softmax computation layer for the label prediction using BN with a dropout rate of 0.5. The CNN model was constructed with 3 variants of word embedding: CNN-random, CNN-fixed-Word2Vec, and CNN-Word2Vec. The only difference is that the parameters of the embedding part were randomly initialized, transferred from pretrained SGNS Word2Vec, maintained nontrainable, and fine-tuned pretrained SGNS Word2Vec during model training.

#### Long Short-term Memory

**Figure 4:**
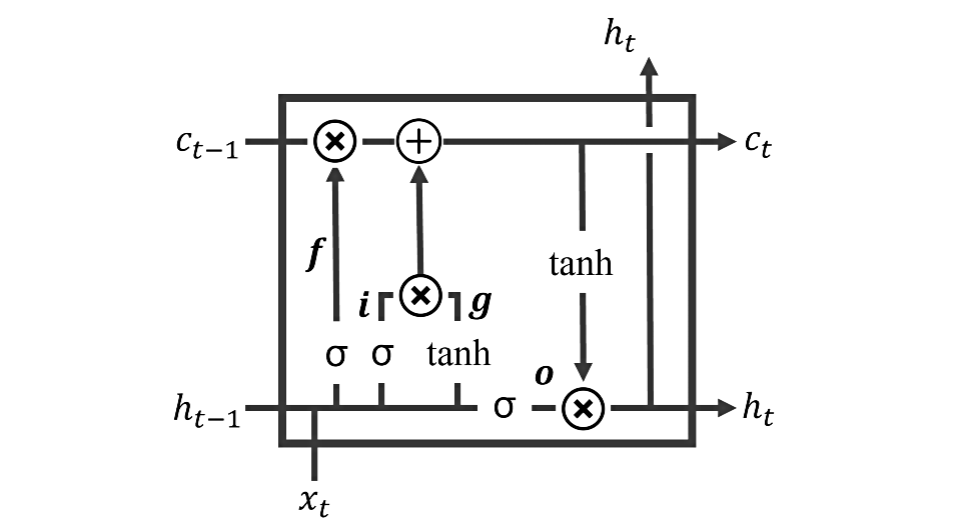
One sample unit of long short-term memory. x: vector; h: hidden state; f: forget gate; i: input gate; g: memory node; o: output gate; c: memory cell; σ: sigmoid function; tanh: hyperbolic tangent function.

RNN can be described as a neural network that learns from sequential data such as time-series data. It has a recurrent structure that learns temporal or sequential patterns and makes the information persistent. However, gradient vanishing is a significant problem while training RNN-based models, and it can cause a long-range dependency when a long input sequence is given. LSTM is a form of RNN structure with added gates in the LSTM interface (Figure 4). Memory cell block alleviates long-term dependency problems. In a unit of LSTM, the forget gate *f_t_*=σ[*W_f_*(*h_t–_*_1_, *x_t_*)+*b_f_*] decides how much to neglect when the previous hidden state and the vector *x_t_* at time *t* are given. The new memory node *g_t_*=tanh[*W_g_*(*h_t–_*_1_, *x_t_*)+*b_g_*] stores new information from the previous hidden state and the vector *x_t_*. The input gate *i_t_*=σ[*W_i_*(*h_t–_*_1_, *x_t_*)+*b_i_*] decides how much new information can be accommodated by element-wise multiplication of the new memory cell. The output gate *o_t_*=σ[*W_o_*(*h_t–_*_1_, *x_t_*)+*b_o_*] determines how much information is delivered to the hidden state *h_t_* at time *t*. In conclusion, the hidden state *h_t_*=*o_t_*·tanh(*c_t_*) is produced with the element-wise multiplication of the output gate and memory cell of *c_t_*, where the memory cell *c_t_*=*f_t_*·*c_t–_*_1_+*i_t_*·*g_t_* is produced by the summation of the element-wise multiplication of the forget gate with previous memory cell of *c_t_*_–1_ and element-wise multiplication of the input gate with the new memory node. σ(*x*)=1/(1*+e^–x^*) is a sigmoid function, tanh(*x*)=(*e^2x^*–1)/(*e^2x^*+1) is a hyperbolic tangent function, and *W* and *b* are distinguishable weight and bias parameters, respectively.

As shown in the middle of Figure 2, for the contextual LSTM model, we constructed 5 unidirectional LSTM layers with 650 units per layer with 2 shortcut connections and a 50% dropout rate on the nonrecurrent connections for every LSTM layer. The bidirectional LSTM model follows the same structure, but each forward and reverse LSTM has 310 units, and 620 units are concatenated per layer. The 2 shortcut connections, which can help prevent the models from overfitting, are linked from the first to the third LSTM layers and from the third to the fifth LSTM layers. We then apply an attention mechanism that can measure the importance of the given tokens before thyroid classification. A hidden representation *u_i_*=tanh(W_u_·h_i_+*b_u_*) from the last hidden layers of LSTM is calculated, and a weighted summation vector *v=*∑*_i_α_i_h_i_* is determined by attention as follows:




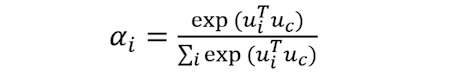




where *W_u_* and *b_u_* are the weight and bias parameters, respectively; and *u_c_* is a context vector that is randomly initialized and jointly learned. The weighted vector then passes to the last layer of this model to compute the softmax probabilities of each thyroid condition. Both Uni-LSTM and Bi-LSTM models vectorize input tokens using the MeCab-ko tokenizer and use trainable pretrained SGNS Word2Vec embedding.

#### Transformer

RNN-based models take a long time to compute input sentences because the calculations are performed sequentially. However, transformer processes input sentences in parallel and capture various relationships between words in a sentence with the help of a multihead self-attention mechanism. Because the input tokens are not computed sequentially, transformer includes special position embedding that reflects position information in the attention mechanism to construct word-to-word importance and dependency. The BERT and ELECTRA models are based on the transformer. The authors of the transformer proposed the architecture of encoder and decoder with a unique attention mechanism. Both BERT and ELECTRA, which are pretrained BERT and ELECTRA, respectively, in our study, use multiple encoder layers of the transformer exclusively, as shown at the bottom of Figure 2. After summing up the 3 tokens, segments, and position embeddings as described above, the transformer encoder obtains linear projections of key, query, and value for each input representation. The scaled dot-product attention is calculated through




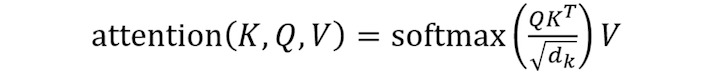




Attention scores are obtained from each query projection by keys, attention weight distribution is computed through a softmax function, and the final values are obtained through the product of the value projection. This attention step is repeated *A*=12 times and concatenated to Concate(head_1_, ..., head_12_)*W^O^* from head*_a_*=attention(*KW_a_^K^*, *QW_a_^Q^*, *VW_a_^V^*), where the dimensions are *d_k_*=*d_q_*=*d_v_*=64, and the distinguishable weights are *W^O^*, *W_a_^K^*, *W_a_^Q^*, and *W_a_^V^*. This can help train the model in which the same input tokens can be represented from multiple perspectives. The results from multihead attention are then connected to 2 layers of feed-forward neural networks FFNN*=*ReLU(0, *xW*_1_+*b*_1_)*W*_2_+b_2_, where the shape of *W*_1_ is (*d*=768, *d_ff_*=3072) and *W_2_* is (*d_ff_*=3072, *d*=768), and processed with residual connection and layer normalization, as depicted with arrows in Figure 2. In conclusion, these encoder layers are stacked 12 times and then connected to the penultimate layer, which is a dense layer with 768 units. Then, the softmax probabilities are computed for predicting the 3 thyroid conditions. The 2 transformer encoder-based models are pretrained with different learning methods: random masking procedures [19] for BERT and replaced token detection [20] for ELECTRA.

### Experimental Settings

The hyperparameters of the different NLP models are listed in Table 4. CNN- and LSTM-based models were trained for 30 epochs with adaptive learning rates by monitoring validation loss; the learning rate decays by a factor of 0.7 if the validation loss is not improved (decreased) within 1 epoch. Transformer encoder–based models, which were initially pretrained with the open-source Korean corpus data, were fine-tuned for 15 epochs with a fixed learning rate. Adam [47] optimizer, where β_1_=0.9, β_2_*=*0.999, and ε=1e–8, is considered for all NLP models. The experiments were implemented with TensorFlow [48], PyTorch [49], and Hugging Face [50] libraries, and a GeForce RTX 2080 Ti 11-GB graphic processor unit.

**Table 4:** Detailed information about different NLP models.

Models	Tokenizer	Embedding vocabulary size	Number of parameters	Initial learning rate	Batch size
Convolution neural network	MeCab-ko	100,000	32 million	1e–3	64
Unidirectional long short-term memory	MeCab-ko	100,000	46 million	2e–4	32
Bidirectional long short-term memory	MeCab-ko	100,000	40 million	2e–4	32
Bidirectional encoder representations from transformers	WordPiece	8002	92 million	2e–5	8
ELECTRA^a^-version 1	WordPiece	32,200	110 million	2e–5	8
ELECTRA-version 2	MeCab-ko & WordPiece	35,000	112 million	2e–5	8

^a^ELECTRA: efficiently learning an encoder that classifies token replacements accurately.

## Results

**Figure 5:**
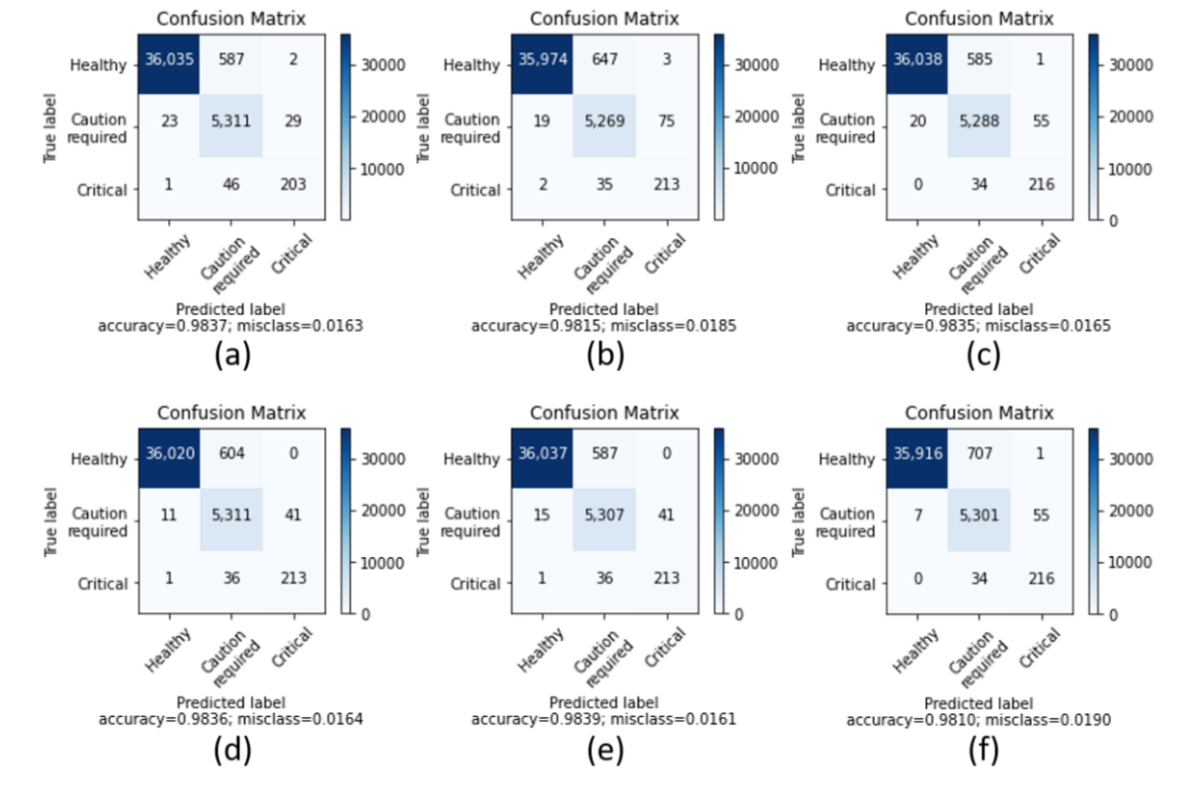
Confusion matrices of multi-label thyroid classification results from the test datasets. All single-architecture-based models are trained or fine-tuned to each ensemble model. The models are (a) CNN-Word2Vec (b) Uni-LSTM (c) ELECTRA-v2 with trimmed data (d) CNN-Word2Vec + Uni-LSTM + ELECTRA-v2 with trimmed data (e) SCENT-v1 (f) SCENT-v2.

According to Table 5 and Figure 5, the macroaveraged precision, recall, and F1 scores are calculated due to the imbalance of multilabel data sets and confusion matrices, respectively. For the single-architecture–based ensemble models, in general, we observed that CNN-Word2Vec achieved the highest F1 score among the ensemble models, and Uni-LSTM outperformed Bi-LSTM by achieving slightly higher F1 scores. Performance degradation was observed in the CNN-Word2Vec and Uni-LSTM models while training with trimmed data sets, but improvement was observed in ELECTRA-v2. The LSTM architecture has the characteristics of an RNN, and it has connections between units along a temporal sequence. Thus, we assume that there must be a difficulty in learning contextual representations owing to the inconsistency of the data structure: lists of words or phrases and full complete sentences. Although both BERT and ELECTRA models have recorded state-of-the-art results on multiple NLP benchmarks, it is surprising that fine-tuned transformer encoder layer–based models do not achieve the highest F1 score in this classification task even with the highest number of parameters. This is likely because there must be an information loss by input sequence truncation even after the keyword-based trimming method or quality issues about these data sets themselves and the data clearing part.

**Table 5:** Experimental results from different NLP models. The test results are macroaverage classification values.

Methods (model name) and models		Precision (%)	Recall (%)	F1 score (%)
**Convolution neural network** **(CNN)**				
	CNN-random^a^	89.33	90.67	89.91
	CNN-fixed-Word2Vec^b^	88.01	93.12	90.43
	CNN-Word2Vec^c^	92.01	92.87	92.33
**Long short-term memory**				
	Unidirectional long short-term memory	87.23	93.89	90.32
	Bidirectional long short-term memory	87.97	92.48	90.09
**Transformer encoder**				
	Bidirectional encoder representations from transformers	86.44	89.69	87.99
	ELECTRA^d^-version 1	87.73	92.12	89.82
	ELECTRA-version 2	91.03	92.33	91.60
**Data trimming**				
	CNN-Word2Vec (trimmed^e^)	90.59	93.56	91.98
	Unidirectional long short-term memory (trimmed)	84.77	93.30	88.61
	ELECTRA-v2 (trimmed)	89.63	94.47	91.92
**Ensemble** **combination**				
	CNN-Word2Vec + Uni-LSTM	89.53	94.24	91.76
	SCENT^f^-v1: CNN-Word2Vec + ELECTRA-v2 (trimmed)	91.10	94.18	92.56
	Unidirectional long short-term memory + ELECTRA-v2 (trimmed)	89.53	94.24	91.76
	CNN-Word2Vec + unidirectional long short-term memory + ELECTRA-v2 (trimmed)	91.02	94.19	92.52
**Hierarchical ensemble**				
	CNN-Word2Vec and unidirectional long short-term memory + ELECTRA-v2 (trimmed)	91.30	92.86	91.92
	Unidirectional long short-term memory and CNN-Word2Vec + ELECTRA-v2 (trimmed)	86.83	93.88	90.09
	SCENT-v2: ELECTRA-v2 (trimmed) and CNN-Word2Vec + unidirectional long short-term memory	89.04	94.44	91.58

^a^Random: randomly initialized embedding.

^b^Fixed-Word2Vec: nontrainable pretrained Word2Vec embedding.

^c^Word2Vec: trainable pretrained Word2Vec embedding.

^d^ELECTRA: efficiently learning an encoder that classifies token replacements accurately.

^e^Trimmed: data sets are trimmed based on the keyword “thyroid” in the comprehensive medical examination text part.

^f^SCENT: static and contextualized ensemble NLP network.

SCENT-v1 shows the best performance by calculating the average softmax values, or simply soft voting, from the unnormalized prediction logits of the 2 ensemble models among the NLP models. SCENT-v1 results in 0 misclassifications of healthy thyroid conditions under the prediction of critical thyroid conditions. SCENT-v2 substantially reduced the number of misclassifications of caution-required thyroid condition to the minimum under the prediction of healthy thyroid condition while maintaining 0 misclassifications of critical thyroid condition. According to Figure 5, SCENT-v2 records the highest precision value for the “healthy” thyroid condition among all models, including hierarchical ensemble models. In “Hierarchical Ensemble” section of Table 5, the word “and” distinguishes the base model and the combined model. The base model initially classifies the 3 labels and the other combined model reclassifies selected labels where only the base model is predicted as having “healthy” labels.

The classification results based on tokenizing Korean input sequences into subwords with or without morphological analysis by MeCab-ko differ as represented in the transformer encoder section by the variants of ELECTRA. It may be argued that the number of vocabulary sizes is different in ELECTRA-v1 and -v2; however, the WordPiece tokenizer has a strong effect on OOV, and approximately a 2% increase in F1 score is worthy of close attention. The parameters of word embedding are randomly initialized and pretrained from CNN-random and CNN-Word2Vec, and there are increases in the macroaveraged precision, recall, and F1 scores observed from CNN-random to CNN-Word2Vec. This verifies that transfer learning from a pretrained architecture is an effective and convincing technique for developing deep neural network models. Unlike the validation results in which the false negatives for the healthy thyroid condition (Figure 3) are relatively lower in CNN-Word2Vec and Uni-LSTM ensemble models, the numbers of false negatives from the CNN-Word2Vec, Uni-LSTM, and ELECTRA-v2 (trimmed) ensemble models in the test data sets do not differ. The false positives from the ELECTRA-v2 (trimmed) ensemble model were still lower than those from the other ensemble models. Overall, all ensemble models, including SCENT-v1 and SCENT-v2, showed poor performance in classifying healthy thyroid conditions under the prediction of the caution-required thyroid condition data sets.

## Discussion

### Limitations

The experiments were originally intended to use only the medical results of the individual thyroid diagnoses. However, the full results of individual text diagnosis of thyroid diseases with hormone examination results and comprehensive medical examination text reports, including doctors’ comments, simple body checkups, health care–related guides, and so on, are used as inputs of the models to reduce human curation as much as possible. If the results are labeled as healthy, the keyword “normal” may be mentioned in the reports. In some cases, the results of the examination, which are supposed to be classified as caution required, are labeled as healthy based on the phrase “no change” compared with reports of previous years (1 or 2 years). This can be one of the reasons as to why the number of misclassifications does not dramatically decrease in every experimental model. Furthermore, it cannot guarantee that data clearing was perfectly conducted over the entire nonstandardized 122,581 data sets from 284 health care institutions. It is highly expected that systematic improvement of data quality may enhance all models’ performance.

The amount of information in each data varies, and individual or comprehensive finding reports cannot be directly used as a single unit during manual classification. Accordingly, the final decisions were concluded by considering all the data sets. The comprehensive text reports may contain information about thyroid tests regardless of the flow in context, and some are typed manually on a case-by-case basis or automatically filled by enumerating predefined text phrases or sentences depending on the institutions and medical professionals, such as sample data of healthy and caution-required thyroid conditions in Table 2. Depending on the experts, the selection and order of predefined texts may differ for the same thyroid diagnosis. This is partly considered advantageous in deciding thyroid classification by only considering numerous static word representations rather than full contextual word representations and their relationships based on such fragmentary compositions of keywords or phrases in the data sets. This can be a reason as why the single-architecture–based CNN ensemble model achieves the highest F1 score compared with other single-architecture ensemble models of Uni-LSTM and ELECTRA-v2 with trimmed data. However, both contextual models recorded higher recall scores than the static model.

Trimming sentences based on the keyword “thyroid” in comprehensive examination reports because of the limitation of 512 tokens shows an improvement in recall and F1-scores in the ELECTRA-v2 ensemble model. This simple preprocessing, however, cannot guarantee whether the optimal data corresponding to thyroid ultrasonography are used as inputs. We find that the improvement in ELECTRA-v2 indicates that preparing a more suitable data set is meaningful under the sequence length limitations. It is highly expected that the performance of the ELECTRA ensemble model can be further enhanced if the limitation is addressed, and the thyroid ultrasound–related contents can be accurately summarized from comprehensive examination reports. However, performance degradation was observed in the CNN-Word2Vec and Uni-LSTM ensemble models when the same trimming procedure was conducted. This proves that other examination reports in addition to thyroid ultrasound data may have valuable information that can help in the classification of thyroid conditions. This allows us to assume that the decline in health conditions caused by thyroid disease can have an effect related to a person’s physical and biological vitality.

### Conclusions and Future Research

Our SCENT models show meaningful results despite the lack of data, especially for the critical condition and unique characteristics of Korean, such as auxiliary, adverbial case markers, and word spacing inconsistency. Additionally, our ensemble model methodologies can be applied to data sets with diverse languages and different sequence lengths if only the WordPiece tokenizer is used. Our SCENT models can not only automate the classification of large-scale text data sets at a high speed while maintaining multiclassification performance, but also reduce the human labor force. For SCENT-v1, misclassifying the “critical” case as “caution required” is much less damaging than misclassifying it as “healthy” in this study. However, this model cannot be directly adopted in real-life applications because both type 1 and 2 errors must be considered. Specifically, the false-positive errors under the prediction of caution-required thyroid conditions are too high to be used.

To consider SCENT models for practical use, we preferentially aim to correctly predict the healthy condition labels, which constitute the largest portion among the 3-class data sets. The model SCENT-v2, which is a hierarchical ensemble of CNN-Word2Vec, Uni-LSTM, and ELECTRA-v2 with trimmed data ensemble models, can reduce the number of incorrect classifications of caution-required condition data to a minimum compared with other approaches, while maintaining the number of misclassified critical condition data set to 0 under the healthy thyroid condition prediction. For further studies, the receiver operating characteristic (ROC) and area under the curve (AUC) algorithms, or simply the AUC–ROC curve, can be considered. For the healthy (negative) thyroid classification, the best or optimal threshold value for the classifier based on rest (positive) conditions can be calculated for suitable healthy thyroid prediction performance. Furthermore, as discussed above, the keyword-based trimming method shows that incorporating additional medical results, which are relevant to disease diagnosis and other physical examinations, may enable us to build classification models to outperform the current models that consider only selected examination results: individual text diagnosis of thyroid diseases, hormone examination results, and comprehensive medical examination text reports, including doctors’ comments. We may also consider developing DL models that can reflect the results derived from the existing interdisease correlation study [51-53] or causality study [54-57].

## Abbreviations

AUC: area under the curve
BERT: bidirectional encoder representations from transformers
BN: batch normalization
CBOW: continuous bag of words
CNN: convolution neural network
DL: deep learning
ELECTRA: efficiently learning an encoder that classifies token replacements accurately
EMR: electronic medical record
LSTM: long short-term memory
NLP: natural language processing
OOV: out of vocabulary
RNN: recurrent neural network
ROC: receiver operating characteristic
SCENT: static and contextualized ensemble NLP network
SGNS: skip-gram with negative sampling
